# Presence of Roe Deer Affects the Occurrence of *Anaplasma phagocytophilum* Ecotypes in Questing *Ixodes ricinus* in Different Habitat Types of Central Europe

**DOI:** 10.3390/ijerph16234725

**Published:** 2019-11-27

**Authors:** Zuzana Hamšíková, Cornelia Silaghi, Katsuhisa Takumi, Ivo Rudolf, Kristyna Gunár, Hein Sprong, Mária Kazimírová

**Affiliations:** 1 Institute of Zoology, Slovak Academy of Sciences, Dúbravská cesta 9, 84506 Bratislava, Slovakia; svitalkova@gmail.com; 2 Institute of Infectology, Friedrich-Loeffler-Institute, Südufer 10, 17493 Greifswald-Insel Riems, Germany; Cornelia.Silaghi@fli.de; 3 Laboratory for Zoonoses and Environmental Microbiology, National Institute for Public Health and Environment, Antonie van Leeuwenhoeklaan 9, P.O. Box 1, 3720 BA Bilthoven, The Netherlands; katsuhisa.takumi@rivm.nl (K.T.); hein.sprong@rivm.nl (H.S.); 4 Institute of Vertebrate Biology, The Czech Academy of Sciences, Klášterní 2, 69142 Valtice, Czech Republic; rudolf@ivb.cz; 5 Institute of Macromolecular Chemistry, The Czech Academy of Sciences, Heyrovského nám. 1888/2, 16200 Prague 6, Czech Republic; venclikova@imc.cas.cz

**Keywords:** *Anaplasma phagocytophilum*, ecotype, *Ixodes ricinus*, deer

## Abstract

The way in which European genetic variants of *Anaplasma phagocytophilum* circulate in their natural foci and which variants cause disease in humans or livestock remains thus far unclear. Red deer and roe deer are suggested to be reservoirs for some European *A. phagocytophilum* strains, and *Ixodes ricinus* is their principal vector. Based on *groEL* gene sequences, five *A. phagocytophilum* ecotypes have been identified. Ecotype I is associated with the broadest host range, including strains that cause disease in domestic animals and humans. Ecotype II is associated with roe deer and does not include zoonotic strains. In the present study, questing *I. ricinus* were collected in urban, pasture, and natural habitats in the Czech Republic, Germany, and Slovakia. A fragment of the *msp2* gene of *A. phagocytophilum* was amplified by real-time PCR in DNA isolated from ticks. Positive samples were further analyzed by nested PCRs targeting fragments of the *16S* rRNA and *groEL* genes, followed by sequencing. Samples were stratified according to the presence/absence of roe deer at the sampling sites. Geographic origin, habitat, and tick stage were also considered. The probability that *A. phagocytophilum* is a particular ecotype was estimated by a generalized linear model. *Anaplasma phagocytophilum* was identified by genetic typing in 274 *I. ricinus* ticks. The majority belonged to ecotype I (63.9%), 28.5% were ecotype II, and both ecotypes were identified in 7.7% of ticks. Ecotype II was more frequently identified in ticks originating from a site with presence of roe deer, whereas ecotype I was more frequent in adult ticks than in nymphs. Models taking into account the country-specific, site-specific, and habitat-specific aspects did not improve the goodness of the fit. Thus, roe deer presence in a certain site and the tick developmental stage are suggested to be the two factors consistently influencing the occurrence of a particular *A. phagocytophilum* ecotype in a positive *I. ricinus* tick.

## 1. Introduction

*Anaplasma phagocytophilum* is a tick-borne obligate intracellular bacterium causing anaplasmosis in humans and animals. Its ecology and epidemiology are very complex in Europe due to the occurrence of several ecotypes that circulate in distinct transmission cycles [1,2,3,4,5]. By using molecular markers, the presence of diverse *A. phagocytophilum* genetic variants has been reported in a wide range of free-living and domestic animals [6,7,8,9,10]. Cervids can be reservoirs for several *A. phagocytophilum* genetic variants transmitted by *Ixodes ricinus*. Certain variants associated with roe deer (*Capreolus capreolus*) are probably nonpathogenic to humans, domestic ruminants, horses, and dogs, while red deer (*Cervus elaphus*) are likely to be reservoirs for variants pathogenic to humans, domestic ruminants, and horses [3,11,12,13,14]. However, co-infection of roe deer with two–three distinct genetic variants, including those causing disease in domestic ruminants, has also been reported recently [15]. The potential reservoir role of wild boar (*Sus scrofa*) for the human granulocytic agent has been suggested [16,17], but generally the role of free-living animals in circulation of *A. phagocytophilum* strains of medical and veterinary importance is still incompletely understood.

Based on *groEL* gene sequences derived from vertebrates and ticks, four or five *A. phagocytophilum* ecotypes have been identified, each associated with different host ranges in Europe [3,18]. The majority of the analyzed sequences belonged to ecotype I and II. Members of ecotype I infect the broadest host range, including wild-living ungulates and *I. ricinus* ticks, and comprise also strains causing disease in domestic animals and humans. Ecotype II was found to be associated with roe deer and *I. ricinus* ticks and does not appear to include zoonotic strains. Ecotype III was identified only in rodents and the rodent-associated *Ixodes trianguliceps* ticks, and ecotype IV was found to be associated with birds and ticks feeding on birds (mainly *Ixodes frontalis*, but also *I. ricinus*) [3]. Ecotype V was identified in *Ixodes ventalloi* ticks from southern Europe, but its pathogenic potential is thus far unknown [18]. Although classification of ecotypes based on the *groEL* gene sequences was found to be more clear than classification based on the *16S* rRNA gene sequences, still more research is required to elucidate the epidemiological significance of European *A. phagocytophilum* genetic linages and ecotypes [4,5,10].

The main objective of the present work was to investigate which factor(s) out of geographic location, habitat type, presence/absence of roe deer, or tick developmental stage could determine the presence of particular *A. phagocytophilum* ecotypes in questing *I. ricinus* ticks.

## 2. Materials and Methods

### 2.1. Study Sites

Questing *I. ricinus* ticks were collected in different habitats—urban, pasture, and natural, with regard to the occurrence of various potential reservoir hosts for *A. phagocytophilum*—in three countries of Central Europe: Czech Republic, Germany, and Slovakia (Table 1).

Six study areas were selected in Bavaria, southern Germany (see [19,20,21] for detailed descriptions of sites):

**A1.**  The southern part of Englischer Garten is a city center park in Munich under strong anthropogenic influence, highly visited by citizens and dogs, maintained by gardening. Interaction between domestic and free-living animals is limited to mice, hedgehogs, foxes, and birds.
**A2.**  Nymphenburger Schlosspark is a city park in Munich, surrounded by walls. Several forest-like structures with higher tree density, scrub, and free-living animals such as roe deer are present in this park. Grassy areas are extensively used and less frequently mowed.
**A3.**  Dörnbergpark is situated in the city center of Regensburg. It is a small park, surrounded by walls, with strong anthropogenic influence, which is expressed by a high frequency of visitors spending their leisure time there. The site is a well-tended park with mostly grassy landscape and only a few old trees such as oaks and maples. Large free-living mammals like roe deer and wild boar are not present.
**A4.**  Schlosspark Berg is a forest-like park on the eastern shore of Lake Starnberg with scrub and walkways. Deciduous trees and bushes, large free-living mammals such as roe deer and foxes exist and hunting is practiced.
**A5.**  Kerschlach is an agricultural site formed by two fenced pastures, which are used for stock breeding of cattle, and is surrounded by a forest area. Contact between wild animals (also roe deer) and domestic cattle exists because animals from the forest can enter the pasture.
**A6.**  Tussenhausen (Angelberger Forst) is a large mixed forest with low anthropogenic influence. This forest is mainly dominated by beech, oak, and spruce. Different free-living animal species are present, and the frequency of visitors is low.

Study sites in the Czech Republic were located in five areas and were characterized in detail in [22,23]:

**B1.**  Valtice is an enclosed, fenced urban park in the castle grounds, where vegetation is periodically adjusted. The fauna is represented by birds, small mammals, and roe deer, the vegetation by broad-leaved deciduous trees and mown grass.
**B2.**  Pohansko is a natural floodplain forest. The fauna consists of birds, various small and large mammals such as roe, red and fallow deer, and wild boar, the vegetation of deciduous forests.
**B3.**  Suchovské mlýny is a pasture area; ticks were sampled along a sheep fence. The fauna is represented by birds, small mammals, and sheep, the vegetation by some trees and shrubs.
**B4.**  Proskovice is a natural ecosystem outside the town of Ostrava. This mixed forest with dominant broad-leaved trees is rarely visited by people. The fauna consists of small and medium-sized mammals, roe deer, birds, and occasionally wild boar.
**B5.**  Bělský les is an urban park in Ostrava. The local fauna is represented by birds and small mammals, and the vegetation by broad-leaved deciduous trees and grass. The park is surrounded by housing estates and used for leisure activities and dog-walking.

In **Slovakia,** four study sites were selected (for more details see [24,25]):

**C1.**  The campus of the Slovak Academy of Sciences (SAS) is an enclosed urban area, within the built-up plots there are patches of broad-leaved forest vegetation, shrubby plots, mown and unmown grassy plots. The fauna consists mainly of birds, feral cats, hedgehogs, and roe deer. The density of rodents is very low.
**C2.**  Mokrohájska street is an urban area situated along fences of gardens and cottages in the vicinity of the SAS campus. The fauna is represented by birds, small mammals, and lizards.
**C3.**  Železná studienka is part of Bratislava Forest Park, located on the foothills of the Small Carpathians Mountains in the northern part of Bratislava. The vegetation is represented by broad-leaved trees, the fauna by birds, small and medium-sized mammals, deer, and wild boar. Železná studienka serves for relaxation of inhabitants of Bratislava and dog walking.
**C4.**  Fúgelka is a natural forest site visited by tourists, rangers, and hunters. The vegetation is represented by a mixed forest, the fauna by various small, medium-sized, and large free-living mammals such as roe, red and fallow deer, mouflon, and wild boar.

### 2.2. Tick Sampling, DNA Extraction, and PCR Amplification

Questing *I. ricinus* ticks were collected from vegetation during previous studies carried out in Bavaria (Germany) in 2011–2012 [20], in eastern and south-eastern Czech Republic in 2010–2014 [22,23], and in south-western Slovakia in 2011–2013 [24].

In each laboratory, DNA was extracted from individual nymphal and adult ticks by commercial isolation kits. A 77-bp long fragment of the *msp2* gene of *A. phagocytophilum* was amplified by real-time PCR, according to Courtney et al. [26] and as described in Overzier et al. [20], Venclikova et al. [22,23], and Svitálková et al. [24], for ticks from Germany, the Czech Republic, and Slovakia, respectively. To identify *A. phagocytophilum* variants, positive samples with Ct values ≤30 were further analyzed with a nested PCR targeting a 497-bp fragment of the *16S* rRNA gene [27], as described in Overzier et al. [20], and with primers targeting a fragment of the *groEL* gene, according to Alberti et al. [28]. Primers and probes used in the PCR reactions are listed in Table 2. All sequences were confirmed by sequencing of both strands.

### 2.3. Sequence Analysis

*16S* rRNA gene sequences were analyzed by ChromasLite^®^ (Technelysium Pty Ltd., South Brisbane, Australia) and by MEGA 6 [29] and aligned with MUSCLE [30]. Database searches and sequence comparisons were performed with the BLAST tool provided by the National Centre for Biotechnology (http://blast.ncbi.nlm.nih.gov/Blast.cgi). The *16S* rRNA gene variants were designated according to Schorn et al. [31] and Silaghi et al. [32]. *GroEL* gene sequences were analyzed in Bionumerics (Version 7.5, Applied Math, Sint-Martens-Latem, Belgium), after subtraction of the primer sequences.

### 2.4. Statistical Analyses

*Anaplasma phagocytophilum* ecotypes were assigned to each sequence, as described previously [3]. Samples were stratified according to the presence and absence of roe deer at the sampling locations. Only those samples for which amplicons could be obtained for both the partial *16S* rRNA and the *groEL* gene were included in the analysis. Next to this, other factors such as geographic origin (country), landscape, and the different *I. ricinus* tick developmental stages were taken into account (Table 3). Each *A. phagocytophilum* ecotype identified in the *I. ricinus* ticks was recorded once into a database for this analysis. As genetic typing was performed at the *16S* and *groEL* loci, sometimes both ecotype I and II were identified in a single *I. ricinus* tick. We consider these ticks to be co-infected with two different *A. phagocytophilum* ecotypes.

We estimated the probability that *A. phagocytophilum* is a particular ecotype by fitting a generalized linear model (glm) to the resulting database, assuming binomial distribution and a logit link. The best-fit model was identified by running the command “step” in forward and backward directions. Collinearity was identified by calculating the condition number of the model matrix. All computations were performed using R [33].

A chi-square (*χ*^2^) test of independence was performed by using PAST [34] to examine the relationship between presence/absence of roe deer and the occurrence of ticks infected with particular *16S* rRNA gene variants.

## 3. Results

*Anaplasma phagocytophilum* ecotypes were identified by genetic typing in a total of 274 infected *I. ricinus* ticks that were collected in urban, suburban, park, pasture, and natural landscapes at 13 geographically distinct sites in three Central European countries (Table 1). In the majority of those samples, only one ecotype was identified: ecotype I (*n* = 175; 63.9%); ecotype II (*n* = 78; 28.5%). Both ecotype I and ecotype II were identified in 21 (7.7%) *I. ricinus* samples (Table 3; Supplementary file 1: Table S1).

A model taking into account roe deer presence in the sampling sites and nymphal and adult stages of ticks fitted best to the dataset, as judged by the minimal value of Bayesian Information Criterion. Ecotype II was more frequently identified in an *I. ricinus* tick when it was collected in a territory with presence of roe deer (*p* < 0.001). Ecotype I was more frequently identified in adult *I. ricinus* ticks compared with nymphs (*p* = 0.004). Alternative models taking additionally into account the country-specific, site-specific, and landscape-specific aspects in our dataset did not improve the goodness of the fit.

Akaike Information Criterion (AIC) is an alternative measure for the goodness of fit. Based on AIC, roe deer and tick’s developmental stage remained significant factors, but sampling site emerged as a new significant factor. None of the individual estimates for a specific site were significant. This led to an extra assessment of the new factor. Roe deer, tick stage, and site were collinear, as detected by the condition number (=62) of the model matrix. High collinearity and insignificant parameter estimates for individual sites led us to conclude that roe deer presence and tick’s developmental stage are the only two factors consistently influencing the occurrence of a particular ecotype in an *A. phagocytophilum-*positive *I. ricinus* tick.

By analyzing *16S* rRNA gene sequences obtained from 291 ticks (the number of ticks for which amplification of the *16S* rRNA fragment was successful was higher than for those for which both the *16S* rRNA and the *groEL* fragments could be amplified), 13 *A. phagocytophilum* variants were identified. Their occurrence and frequency varied between the study sites (Table 4). Variant A was the most frequent (59.1%). The overall proportion of ticks infected with variant A was significantly related to the presence of roe deer (*χ*^2^ = 64.146, *p* < 0.001) and was higher in sites with absence of these ungulates (90.3% vs. 42.0% in sites with roe deer) (Table 4). This trend was similar in both nymphs and adult ticks (nymphs: *χ*^2^ = 8.579, *p* < 0.01; adults: *χ*^2^ = 54.307, *p* < 0.001). In sites where roe deer were present, ticks carrying variant X (total: *χ*^2^ = 21.228, *p* < 0.001; nymphs: *χ*^2^ = 8.811, *p* < 0.01; adults: *χ*^2^ = 10.896, *p* < 0.001) and Y (total: *χ*^2^ = 11.155, *p* < 0.01; nymphs: *χ*^2^ = 0.617, ns; adults: *χ*^2^ = 11.379, *p* < 0.001) occurred more frequently than in sites without deer (Table 4).

## 4. Discussion

*Ixodes ricinus* is a generalist three-host tick that is able to parasitize a wide range of vertebrate hosts and transmit a great variability of microorganisms, including *A. phagocytophilum* [35]. The ecology of *A. phagocytophilum* in Europe is very complex and involves specific host–microbe–tick associations and distinct transmission cycles in which zoonotic and nonzoonotic strains circulate [2]. In spite of existing knowledge on associations of particular European *A. phagocytophilum* genetic lineages to certain vertebrate hosts and vector ticks and on their pathogenicity to humans and domestic animals, the underlying mechanisms determining the genetic variability of the bacterium are still not clear. *Ixodes ricinus,* due to its complex life cycle and wide host range, but mainly vertebrate reservoirs, due to peculiarities of their immune system, seem to drive the evolution and distribution of *A. phagocytophilum* genetic lineages [5].

It has already been confirmed that roe deer are important feeding hosts for all developmental stages of *I. ricinus* and reservoirs for particular nonzoonotic genetic variants of *A. phagocytophilum* [3,12,32,36,37], whereby the overall prevalence of infection in roe deer in particular sites can be higher than 90% [36,37,38,39]. The main objective of this study was to find out if, and to what extent, presence/absence of roe deer in a particular site can determine the occurrence of *A. phagocytophilum* strains in questing *I. ricinus* ticks. For this purpose, 13 sites located in three European countries and different landscapes were selected for tick collection, and the *A. phagocytophilum* strains in ticks were characterized based on partial *16S* rRNA and *groEL* gene sequences. *Anaplasma phagocytophilum* was previously detected by PCR in questing *I. ricinus* ticks from each of the study sites, with total prevalence ranging between 1.4% and 19.7% (see Table 5). However, presence/absence of roe deer did not seem to affect the overall infection rates, except for an urban fenced area in Bratislava (Slovakia) with a relatively high roe deer density and a 19.7% *A. phagocytophilum* prevalence (Table 5 and [24]). Because transovarial transmission has not been confirmed in ticks, different reservoir hosts are likely to maintain the circulation of the bacterium in sites without roe deer presence. It was previously indicated for particular regions that the distribution of *A. phagocytophilum* ecotypes in ticks was linked to the presence of certain cervid species [20,36,37,40]. The prevalence of the roe deer-associated ecotype II as well as of the *16S* rRNA variants X and Y (ranked among ecotype II, see Supplementary file 1: Table S1) in questing *I. ricinus* nymphs and adults was indeed higher in our study sites with roe deer presence than in locations where roe deer were absent. However, infection rates in adult ticks and nymphs differed in those sites: ecotype II was detected in 54.2% of nymphs and 38.0% of adults, *16S* rRNA variant X occurred in 34.6% of nymphs and 14.7% of adults, and *16S* rRNA variant Y was detected in 11.5% of nymphs and 12.5% of adults. These differences are difficult to explain, but could be, for example, due to nymph mortality, dispersal to other locations, or diminishing of the infection during molting to the adult stage. Transstadial transmission of some *A. phagocytophilum* strains has been confirmed experimentally [41,42], but transmission rates for the roe deer-associated strains are unknown. It is also not clear if and to what extent the infection with a particular *A. phagocytophilum* strain perpetuates in adult ticks in case infected nymphs feed on uninfected or noncompetent hosts or feed on hosts infected with other *A. phagocytophilum* strains or other pathogen(s). For example, in an agricultural site in France with high cattle abundance and about 80% *A. phagocytophilum* prevalence in roe deer, cattle density was suggested to be the main factor affecting the bacterial prevalence and occurrence of genotypes in questing *I. ricinus,* because almost 80% of the examined ticks carried sequences associated with infections in cattle and humans, and the high infection prevalence in roe deer was probably due to superinfections [43].

Ticks harboring *A. phagocytophilum* ecotype I that comprises strains associated with a wide range of different mammal species including red deer, small free-living and domestic ruminants, wild boar, carnivores, hedgehogs, and humans, were found to prevail in both types of sites, that is, with presence/absence of roe deer, but the infection rates were higher in sites without roe deer. This suggests that in sites with presence of roe deer the transmission hosts are not limited only to these animals but include a wider spectrum of vertebrate species, whereas in sites without roe deer, the bacterium is maintained by other reservoir hosts. The occurrence of ticks infected with ecotype II in sites where roe deer were absent may be the result of transport of those ticks by birds or mammals with large home ranges.

In total, about 7% of ticks (nymphs as well as adults) from the two types of sites harbored both ecotype I and II, suggesting that either the ticks fed on hosts co-infected with different *A. phagocytophilum* strains or larvae and nymphs fed successively on hosts harboring various strains. Simultaneous infection of vertebrates with multiple *A. phagocytophilum* strains was found to exist and has recently been confirmed, for example, for roe deer from France that harbored 2–3 *ankA* gene variants [15] or cattle from Germany infected with two *ankA* variants and multiple MLST sequence types [44]. Co-infections may lead to bacterial recombination and emergence of new genetic variants [44,45]. However, it is not clear if multiple strains are co-transmitted by *I. ricinus.*

The rodent-associated ecotype III and the bird-associated ecotype IV were not found in this study, although presence of rodents and birds was observed in the majority of the study sites. The main reasons why these ecotypes were not detected are probably because (1) the representation of ecotypes III and IV is generally much lower than that of ecotypes I and II [3], and (2) only questing *I. ricinus* were analyzed, while the vector of ecotype III is the endophilic *I. trianguliceps* tick and ecotype IV can be found mainly in birds and bird-feeding *I. frontalis* and *I. ricinus* ticks.

Analysis of the variability of the partial *16S* rRNA gene revealed the presence of 13 *A. phagocytophilum* variants in *I. ricinus*: nine, six, and ten from sites in Germany, the Czech Republic, and Slovakia, respectively (see Table 4). In addition to the roe deer-associated variants X and Y (discussed above), the occurrence and prevalence of the other variants also differed in the study sites, whereby their spectrum was generally wider in natural sites with occurrence of roe deer and numerous free-living vertebrate host species than in urban areas and pastures. Although a basic distinction of variants associated with free-living ruminants, domestic animals, or both groups can be achieved with *16S* rRNA gene-based typing [32,37], this approach is not reliable to define *A. phagocytophilum* genotypes [12,14,36,44]. The occurrence of *16S* rRNA variants in questing *I. ricinus* ticks and roe deer, their possible pathogenicity to domestic ruminants and humans, and their association with free-living reservoir hosts in the study sites in Germany have extensively been discussed in earlier publications [20,31,37]. However, the results obtained for sites in the Czech Republic and Slovakia are new. Based on analysis of a limited number of free-living ungulates and engorged ticks from the Small Carpathian Mountains in south-western Slovakia, where the forest park and natural tick collection site are located, variants Y and X (ecotype II) were previously detected in roe deer and roe deer attached ticks, respectively. Ecotype I, comprising variant B, was detected in wild boar and fallow deer attached ticks, variant S in fallow deer, variant S and W in red deer and fallow deer attached ticks, and variant W in mouflon [39]. These variants were also detected in questing ticks in our study and in addition, variants A, I, J, N, and V were identified, whereby variants I, J. N, V and some sequences of variant W were ranked among ecotype II (see Supplementary file 1: Table S1). Both variant A (ecotype I) and V (ecotype II) were previously found associated with, for example, questing *I. ricinus* ticks and hedgehogs, while variant A was also associated with red fox, cat, dog, horse, and a human case [36], and variant V was detected in roe deer, fallow deer, wild boar, rodents, and small ruminants [20,31,46,47,48]. Similar to Germany, variant A prevailed in the Slovak urban site without roe deer. Interestingly and in contrast to Germany, a relatively high proportion of ticks carrying variant A was found in Slovak suburban and natural sites with roe deer presence (see Table 4). This suggests that the host range of this variant may be wider than previously thought, and the spectrum and abundance of potential reservoir hosts differ between the sites in the two countries. Variant B, which is identical with the prototype human pathogenic variant of *A. phagocytophilum*, was detected only in one adult tick from the natural site, but was previously identified in wild boar from the same area [39] and also in horses and dogs with granulocytic anaplasmosis in Germany [47,48]. Variants I and J were identified, for example, in roe deer and red deer in Germany and Austria, respectively [32,37], and variant N in roe deer from Germany [37]. Variant S was found, for example, in red deer, roe deer, and ibex in Austria [32] and in red deer and fallow deer in the Slovak study area [39]. Variant W was found to be associated with clinical cases of tick-borne fewer in domestic ruminants, but was identified also, for example, in roe deer, red deer, chamois, and mouflon [32,37,39].

To our knowledge, there are no data on occurrence of *16S* rRNA variants or ecotypes based on *groEL* sequences in vertebrate hosts from the study sites in the Czech Republic. Similar to Germany and Slovakia, *I. ricinus* ticks with variant A prevailed in the urban park (Bělský les) without roe deer. In the agricultural site Suchovské mlýny, ticks with variant W dominated, suggesting that they probably fed on infected sheep grazing in that area. Interestingly and in contrast to German and Slovak sites, ticks harboring variant A dominated in sites with roe deer presence, and only 16.7% of ticks carried roe deer-associated variants X and Y. Presence of variant B was confirmed in ticks from the urban park in Valtice and in the natural site Pohansko. The occurrence of variants B and W suggests that *I. ricinus* is also involved in the circulation of *A. phagocytophilum* variants pathogenic for domestic animals and humans in the Czech Republic.

## 5. Conclusions

In conclusion, the occurrence and prevalence of *A. phagocytophilum* ecotypes and partial *16S* rRNA gene variants in questing *I. ricinus* ticks from different habitat types of Germany, the Czech Republic, and Slovakia that were investigated during our study depended mainly on the presence/absence of roe deer. In sites with presence of roe deer, the prevalence of ticks infected with nonpathogenic variants of the bacterium was significantly higher than in sites where roe deer were absent. The results suggest that presence of roe deer might, at least to a certain degree, decrease the risk of contracting granulocytic anaplasmosis in domestic animals and humans. However, to support this assumption, further research is needed to elucidate the evolution of *A. phagocytophilum* strains and their associations to vertebrate hosts.

## Figures and Tables

**Table 1:** Geographical location of tick sampling sites.

Country	Acronym	Name of the Site	Landscape	Geographic Coordinates	
Germany	A1	Englischer Garten	Urban park	48°09′01.73″ N	11°35′24.19″ E
	A2	Nymphenburger Schlosspark	Urban park	48°09′38.93″ N	11°29′33.31″ E
	A3	Dörnbergpark	Urban park	49°00′55.72″ N	12°05′08.89″ E
	A4	Schlosspark Berg	Forest park	47°57′43.85″ N	11°20′53.35″ E
	A5	Kerschlach	Agricultural	47°54′57.18″ N	11°12′44.04″ E
	A6	Tussenhausen	Natural	48°06′36.42″ N	10°34′33.40″ E
Czech Republic	B1	Valtice	Urban park	48°44′05.68″ N	16°45′11.31″ E
	B2	Pohansko	Natural	48°43′37.68″ N	16°53′08.35″ E
	B3	Suchovské mlýny	Pastureland	48°53′50.79″ N	17°34′54.94″ E
	B4	Proskovice	Natural	49°44′51.73″ N	18°12′23.45″ E
	B5	Bělský les	Urban park	49°47′5.65″ N	18°14′28.29″ E
Slovakia	C1	SAS campus	Urban park	48°10′14.58″ N	17°04′1.15″ E
	C2	Mokrohájska street	Urban	48°10′34.71″ N	17°04′3.15″ E
	C3	Železná studienka	Forest park	48°12′14.16″ N	17°05′47.05″ E
	C4	Fúgelka	Natural	48°22′44.14″ N	17°18′52.86″ E

**Table 2:** Primers and probes used in PCR reactions for the amplification of *Anaplasma phagocytophilum* gene fragments.

Gene	Primers and Probes (P)	Sequences (5′-3′)	Ref.
*msp2*	ApMSP2f	ATGGAAGGTAGTGTTGGTTATGGTATT	[26]
	ApMSP2r	TTGGTCTTGAAGCGCTCGTA	
	ApMSP2p (P)	HEX-TGGTGCCAGGGTTGAGCTTGAGATTG-TAMRA	
*16S* rRNA	1st amplification:		[27]
	ge3a	CACATGCAAGTCGAACGGATTATTC	
	ge10r	TTCCGTTAAGAAGGATCTAATCTCC	
	2nd amplification:		
	ge9f	AACGGATTATTCTTTATAGCTTGCT	
	ge2	GGCAGTATTAAAAGCAGCTCCAGG	
*groEL*	EphplgroEL-A.phago-F	ATGGTATGCAGTTTGATCGC	[28]
	EphgroEL-A.phago-R	TTGAGTACAGCAACACCACCGGAA	

**Table 3:** Frequency of *Anaplasma phagocytophilum* ecotypes identified in questing *Ixodes ricinus* ticks based on *16S* rRNA and *groEL* gene sequences. In the logit model describing the probability that a particular ecotype is present in an *I. ricinus* tick, the only significant factors are presence/absence of roe deer and tick developmental stage.

Roe Deer	Tick Stage	Country	Site	Landscape	Ecotype I	Ecotype II	Ecotype I & II
Absent	A	Germany	A1	UP	14		
			A3	UP	47		
		Czech	B3	A		1	2
		Republic	B5	UP	1		1
		Slovakia	C2	U	12	1	
	N	Germany	A1	UP	3		
			A3	UP	5		
		Czech	B3	A	1		4
		Republic	B5	UP	4	1	
				SUBTOTAL	87	3	7
				%	89.7	3.1	7.2
Present	A	Germany	A2	UP	8	8	
			A4	FP		5	
			A5	A	2	6	
			A6	N	1	10	
		Czech	B1	UP	5		
		Republic	B2	N	2		1
			B4	N	5		
		Slovakia	C1	U	13	6	1
			C3	FP	15	9	1
			C4	N	19	5	7
	N	Germany	A2	UP	1		
			A6	N		2	
		Czech	B1	UP	2	1	1
		Republic	B4	N	3	2	
		Slovakia	C1	U		7	
			C3	FP	7	9	2
			C4	N	5	5	1
				SUBTOTAL	88	75	14
				%	49.7	42.4	7.9
TOTAL					175	78	21
%					63.9	28.5	7.7

A = adult; N = nymph; for site acronyms see Table 1. UP = urban park, FP = forest park, A = agricultural, pastureland, N = natural forest, U = urban.

**Table 4:** Frequency of *Anaplasma phagocytophilum* variants identified in questing *Ixodes ricinus* ticks based on a fragment of the *16S* rRNA sequence.

Roe Deer	Tick Stage	Country	Site	Landscape		*16S* rRNA Gene Variant											
					A	B	I	J	N	O	P	S	V	W	X	Y	Z
Absent	A	Germany	A1	UP	13						1						
			A3	UP	48												
		Czech	B3	A	1									2	1		
		Republic	B5	UP	4												
		Slovakia	C2	U	13								1				
	N	Germany	A1	UP	3												
			A3	UP	6												
		Czech	B3	A	1									4			
		Republic	B5	UP	4											1	
				SUBTOTAL	93						1		1	6	1	1	
				%	90.3						1.0		1.0	5.8	1.0	1.0	
Present	A	Germany	A2	UP	8								1			7	1
			A4	FP											3	2	
			A5	A		2								1	2	3	
			A6	N		1				1			2	3		4	
		Czech	B1	UP	5												
		Republic	B2	N		1						1			1		
			B4	N	6												
		Slovakia	C1	U	14								1		5		
			C3	FP	15			1	1					2	6	1	
			C4	N	13	1		1				9	1	7	3		
	N	Germany	A2	UP	1												
			A6	N												2	
		Czech	B1	UP	2	1										1	
		Republic	B4	N	4											1	
		Slovakia	C1	U									4		4	1	
			C3	FP	7		2								9		
			C4	N	4							1		2	4	1	
				SUBTOTAL	79	6	2	2	1	1		11	9	15	38	23	1
				%	42.0	3.2	1.1	1.1	0.5	0.5		5.8	4.8	8.0	20.2	12.2	0.5
TOTAL					172	6	2	2	1	1	1	11	10	21	39	24	1
%					59.1	2.1	0.7	0.7	0.3	0.3	0.3	3.8	3.4	7.2	13.4	8.2	0.3

A = adult; N = nymph; for site acronyms, see Table 1. Designation of *16S* rRNA gene variants follows Schorn et al. [31] and Silaghi et al. [32]. UP = urban park, FP = forest park, A = agricultural, pastureland, N = natural forest, U = urban.

**Table 5:** Mean prevalence of *Anaplasma phagocytophilum* in questing *Ixodes ricinus* ticks per site for the study period.

			Roe	Adults		Nymphs		TOTAL	
Country	Site	Landscape	Deer	Positive/Total	%	Positive/Total	%	Positive/Total	%
Germany	A1	UP	-	29/480	6.0	6/238	2.5	35/718	4.9
	A2	UP	+	24/491	4.9	2/260	0.8	26/751	3.5
	A3	UP	-	93/475	19.6	9/240	3.7	102/715	14.3
	A4	FP	+	8/390	2.0	1/226	0.4	9/606	1.5
	A5	A	+	14/459	3.0	0/260	0	14/719	1.9
	A6	N	+	24/305	7.9	4/240	0.8	28/545	5.1
Czech	B1	UP	+	11/178	6.2	6/237	2.5	17/415	4.1
Republic	B2	N	+	3/37	8.1	0/170	0	3/207	1.4
	B3	A	-	5/86	5.8	7/456	1.5	12/542	2.2
	B4	N	+	11/83	13.3	12/1114	1.1	23/1197	1.9
	B5	UP	-	13/96	13.5	13/180	7.2	26/276	9.4
Slovakia	C1	UP	+	139/397	35.0	70/663	10.6	209/1060	19.7
	C2	U	-	23/193	11.9	0/13	0	23/206	11.2
	C3	FP	+	47/344	13.7	32/404	7.9	79/748	10.6
	C4	N	+	30/520	5.8	20/1362	1.5	50/1882	2.7

Data are summarized from publications by Silaghi et al. [19], Overzier et al. [20], Venclikova et al. [22,23], and Svitálková et al. [24]. For site acronyms, see Table 1. UP = urban park, FP = forest park, A = agricultural, pastureland, N = natural forest, U = urban. +/- = presence/absence of roe deer.

## Supplementary Materials

The following are available online at https://www.mdpi.com/1660-4601/16/23/4725/s1, Table S1 List of analyzed *I. ricinus* ticks for *16S* rRNA and *GroEl* genetic variants, including tick developmental stage (N: nymph, F: female, M: male), country of origin, area/site, type of habitat, sampling date, indication of presence/absence of roe deer, and the analyzed *GroEL* and *16S* rRNA sequences.
